# SpoVT: From Fine-Tuning Regulator in *Bacillus subtilis* to Essential Sporulation Protein in *Bacillus cereus*

**DOI:** 10.3389/fmicb.2016.01607

**Published:** 2016-10-13

**Authors:** Robyn T. Eijlander, Siger Holsappel, Anne de Jong, Abhinaba Ghosh, Graham Christie, Oscar P. Kuipers

**Affiliations:** ^1^Top Institute Food and NutritionWageningen, Netherlands; ^2^Department of Molecular Genetics, Groningen Biomolecular Sciences and Biotechnology Institute, University of GroningenGroningen, Netherlands; ^3^Department of Chemical Engineering and Biotechnology, Institute of Biotechnology, University of CambridgeCambridge, UK

**Keywords:** sporulation, germination, gene regulation, *Bacillus cereus*, SpoVT

## Abstract

Sporulation is a highly sophisticated developmental process adopted by most Bacilli as a survival strategy to withstand extreme conditions that normally do not support microbial growth. A complicated regulatory cascade, divided into various stages and taking place in two different compartments of the cell, involves a number of primary and secondary regulator proteins that drive gene expression directed toward the formation and maturation of an endospore. Such regulator proteins are highly conserved among various spore formers. Despite this conservation, both regulatory and phenotypic differences are observed between different species of spore forming bacteria. In this study, we demonstrate that deletion of the regulatory sporulation protein SpoVT results in a severe sporulation defect in *Bacillus cereus*, whereas this is not observed in *Bacillus subtilis*. Although spores are initially formed, the process is stalled at a later stage in development, followed by lysis of the forespore and the mother cell. A transcriptomic investigation of *B. cereus* Δ*spoVT* shows upregulation of genes involved in germination, potentially leading to premature lysis of prespores formed. Additionally, extreme variation in the expression of species-specific genes of unknown function was observed. Introduction of the *B. subtilis* SpoVT protein could partly restore the sporulation defect in the *B. cereus spoVT* mutant strain. The difference in phenotype is thus more than likely explained by differences in promoter targets rather than differences in mode of action of the conserved SpoVT regulator protein. This study stresses that evolutionary variances in regulon members of sporulation regulators can have profound effects on the spore developmental process and that mere protein homology is not a foolproof predictor of similar phenotypes.

## Introduction

Strains of *Bacillus* species are able to form endospores as a survival strategy in response to poor growth conditions. Their metabolic inactive state and sophisticated layered structures lead to strong resistance properties that enable the spore to survive conditions of increased heat, UV radiation, acid concentrations, pressure or low levels of water, or nutrients for extremely long periods of time (Setlow, 2006, 2007; Sella et al., 2014), while maintaining the ability to monitor their surroundings and respond to improvements through the process of germination and outgrowth (Setlow, 2003, 2013; Moir, 2006; Dworkin and Shah, 2010). Sporulation, spore resistance development, spore germination, and spore outgrowth are processes that are characterized by inter-strain and intra-strain heterogeneity and variety. This hampers the eradication of spores from food products or raw ingredients as it complicates predictability of spore properties and behavior (Cronin and Wilkinson, 2008; Augustin, 2011; Eijlander et al., 2011). Returning to a vegetative state, germinated spores are a major cause of food spoilage and of food poisoning (in the case of toxin production) (Brown, 2000; Abee et al., 2011; Logan, 2012).

**Figure 1:**
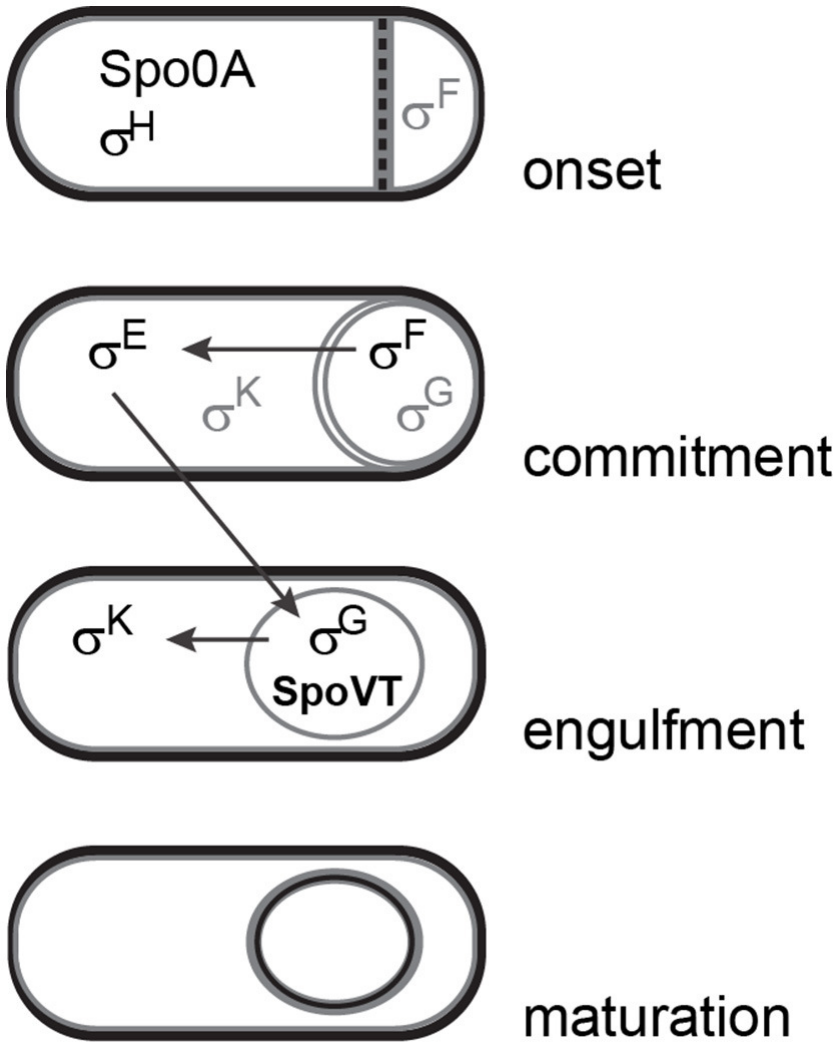
**Simplified schematic overview of different stages in sporulation and sequential activation of sporulation-specific sigma factors**. Gray lettering indicates inactive forms of sigma factors. This figure was adapted from Piggot and Hilbert (2004).

Dormant spores are the final result of sporulation, which involves complex gene regulatory processes taking place in two different compartments of the cell (Eijlander et al., 2014). Sporulation-specific sigma factors govern the expression of dedicated gene sets in various stages, a process that is regulated in a sequential fashion (Hilbert and Piggot, 2004). The early regulator proteins Spo0A and σ^H^ are responsible for the consecutive expression and activation of σ^F^ in the forespore and σ^E^ in the mother cell (Yudkin and Clarkson, 2005). Expression and activation of late-stage sporulation sigma factors σ^G^ in the forespore and σ^K^ in the mother cell depend on completion of these earlier sporulation stages (Li and Piggot, 2001; Chary et al., 2005) (Figure 1). Binding of each sigma factor to RNA polymerase leads to interaction with dedicated DNA targets resulting in the spatial and temporal expression of specific sporulation gene sets required for the development and assembly of the forespore (Rudner and Losick, 2001; Hilbert and Piggot, 2004). Most proteins playing key roles in germination are produced during the later stages in sporulation.

The expression levels of sporulation and germination genes are fine-tuned by secondary regulator proteins that are under the control of sigma factors and enable feed forward loops in the regulatory system. One of these proteins is SpoVT (Figure 1). Expressed in the forespore compartment during late-stage sporulation under the control of σ^G^, SpoVT enhances the expression of some σ^G^-dependent genes and represses others (Bagyan et al., 1996). For DNA binding it forms a tetramer and possibly binds a yet unidentified substrate through means of the GAF domain in the C-terminal part of the protein (Dong et al., 2004; Asen et al., 2009). Deletion of *spoVT* from *B. subtilis* cells results in the formation of spores that have a defective spore coat, a faster nutrient-induced germination response, an increased sensitivity to UV radiation and limited ability for spore outgrowth (Bagyan et al., 1996; Ramirez-Peralta et al., 2012). A recent study in *B. subtilis* has shown that levels of active SpoVT play a crucial role in determining the numbers of important germination proteins, such as nutrient germination receptors (GRs) and small acid-soluble proteins (SASPs), which affects the germination, resistance, and outgrowth properties of the spore (Ramirez-Peralta et al., 2012).

The SpoVT protein is highly conserved amongst spore-forming bacteria, especially *Bacilli* (Asen et al., 2009; Ramirez-Peralta et al., 2012). The extreme sequence similarity (~95%) of the N-terminal domain of SpoVT homologs suggests a similar mode of action concerning DNA binding activity. It must be noted, however, that so far no consensus binding site for SpoVT has been identified (Dong et al., 2004). The C-terminal domain is less conserved (sequence identity of 20–30%) and shows features of a GAF domain that resembles the one of CodY (Asen et al., 2009). Such domains harbor binding pockets for the binding of substrates that are required for structure, sensing, and/or activity. If SpoVT requires substrate-binding for activity, it is possible that this substrate differs amongst various SpoVT homologs (Asen et al., 2009). However, several attempts to identify such a substrate have so far failed.

The SpoVT protein of *Bacillus cereus* (from here on referred to as SpoVT_BCE_) has been less well studied than its *B. subtilis* counterpart and its precise role in gene expression during sporulation is so far unknown. *B. cereus* is notorious for compromising food quality and safety due to its high diversity, the production of highly heat-resistant spores and cytotoxin-producing properties (Stenfors Arnesen et al., 2008; Lücking et al., 2013). It is part of a wider class of related species that also includes *Bacillus anthracis*, the causer of anthrax, and *Bacillus thuringiensis*, an insect pathogen. The main characteristics of these closely related species are determined by large plasmids that encode specific toxin or other virulence factors (Økstad and and Kolstø, 2011). The non-pathogenic *B. subtilis* lacks such plasmids and is part of a different group of Bacilli that also includes *Bacillus licheniformis* and *Bacillus amyloliquefaciens*. Due to a wealth of available experimental data, *B. subtilis* and *B. cereus* are commonly used as model organisms for studies concerning these two groups of species, with in particular *B. subtilis* 168 and *B cereus* ATCC 14579. Despite a common ability to form endospores and a conservation of important sporulation and germination genes (de Vries et al., 2004; de Hoon et al., 2010; Galperin et al., 2012), interesting evolutionary diversity can be observed between these two species. This is reflected by differences in gene regulation during sporulation (Pflughoeft et al., 2011) or in various spore properties, such as the spore coat (Wang et al., 2007; Qin and Driks, 2013), spore size (Carrera et al., 2007), germinant receptor protein types (Hornstra et al., 2005; van der Voort et al., 2010), and spore resistance properties (Wang et al., 2003; Black et al., 2008).

In this study, we investigated the role or SpoVT_BCE_ in (heterogeneous) gene expression during sporulation of *B. cereus*. We show that, in contrast to what was previously reported for *B. subtilis*, deletion of *spoVT*_BCE_ results in a complete sporulation defect. Transcriptomic investigation during sporulation shows upregulation of genes involved in germination, which indicates premature lysis of prespores. In addition, the data shows differential expression of *B. cereus*-specific genes of unknown function, which potentially play an important role in the extreme phenotype. This is furthermore supported by successful complementation studies using both SpoVT_BCE_ and SpoVT_BSU_. Through this study we show that despite strong sequence conservation of SpoVT among Bacilli, significant differences exist in the role of this regulator during developmental processes, which are likely due to the specific genes under its control.

## Materials and methods

### Strains and plasmids

All strains and plasmids used in this study are listed in Table 1. Primers used for the amplification of DNA fragments are listed in Table S1. As reference strains, *B. subtilis* 168 and *B. cereus* ATCC 14579 were used. Foreign DNA was introduced into *B. cereus* via electroporation (Masson et al., 1989), either by using the multicopy plasmid pNW33n or by single crossover DNA integration *via* non-replicative pMAD and PSG1151 derivatives.

**Table 1:** **Strains and plasmids used in this study**.

**Strain**	**Properties**	**References**
*B. subtilis* 168	*trpC2*	Kunst et al., 1997
*B. subtilis* 168 Δ*spoVT*	*spoVT*::*spec^r^*	Bagyan et al., 1996, kindly provided by Prof. S. Cutting
*B. cereus* ATCC 14579	Enterotoxic strain of *B. cereus* wild type isolate	Bacillus Genetic Stock Center, ATCC, BGSC ID6A5
*B. cereus* ATCC 14579 P_*spoVA*_-*gfp*	*P*spoVAA*-gfp*, Cm^r^	This study
*B. cereus* ATCC 14579 Δ*spoVT*	*spoVT*::*spec^r^*	This study
*B. cereus* ATCC 14579 Δ*spoVT* P_*spoVA*_-*gfp*	*spoVT*::*spec^r^, P*spoVAA*-gfp*, Cm^r^	This study
*B. cereus* ATCC 14579 ΔBC1117	marker-less deletion of the BC1117 ORF	This study
**Plasmid**	**Properties**	**References**
pNW33n	*E. coli*–Gram + shuttle vector	Bacillus Genetic Stock Center
pNWVT	Cm^r^, *spoVT*_BCE_	This study
pVTBsu2	Cm^r^, *spoVT*_BSU_	This study
pSG1151	Vector for integrative P-*gfpmut1* fusions in *B. subtilis*, Ap^r^, Cm^r^	Lewis and Marston, 1999
pSGCVA	pSG1151 with *gfp* driven by the *Bce spoVAA* promoter, Ap^r^, Cm^r^	This study
pMAD	Vector for efficient gene replacement in non-naturally transformable gram-positive bacteria, Ap^r^, Em^r^	Arnaud et al., 2004
pDCVT	pMAD-derivative used for *spoVT* gene disruption, Ap^r^, Em^r^	This study
pDG1726	*E. coli* plasmid bearing a spectinomycin resistance cassette	Guérout-Fleury et al., 1995

For the construction of the pNWVT vector, the *spoVT* gene (BC0059) including its own promoter was amplified from *B. cereus* ATCC 14579 chromosomal DNA using primers AKupVT-F and TIFN16. The resulting product was cut with EcoRI and KpnI (Fermentas, FastDigest) and ligated into the corresponding sites of pNW33n (Genbank Accession number, AY237122), which resulted in pNWVT. The pVTBsu2 vector was created by replacing *spoVT*_BCE_ with *spoVT*_BSU_ in pNWVT, which was amplified using primers BsuVTF2 and BsuVTR. The primers were specifically designed to ensure that *spoVT*_BSU_ expression would be driven by the *B. cereus spoVT* promoter already present in pNWVT. The *spoVT*_BCE_ gene was cut out of pNWVT using BclI and HindIII and replaced with *spoVT*_BSU_ containing compatible sticky ends. Correct construction was verified using restriction analysis and sequencing.

For the construction of the *B. cereus spoVT* disruption mutant, we constructed the pDCVT vector by amplifying an upstream *spoVT* flanking region from *B. cereus* ATCC 14579 gDNA using primers dCVT-F1 and dCVT-R1. A downstream flanking region was amplified using primers dCVT-F2 and dCVT-R2. Both flanking regions were fused to HindIII compatible ends of a spectinomycin resistance cassette originating from pDG1726. The fused fragments were ligated into the NcoI and EcoRI sites of pMAD to create pDCVT. The pDCVT was introduced into *B. cereus* ATCC 14579 to disrupt the *spoVT* gene according to the method described by Arnaud et al. (2004). Disruption of *spoVT* was verified using PCR analysis and sequencing of the integration site to ensure there were no second-site mutations.

For the construction of a chromosomally integrated P_*spoVA*_-*gfp* fusion in *B. cereus*, a 1.5 kb fragment of the upstream region of the *B. cereus* ATCC 14579 *spoVAA* gene (BC4070) was amplified using primers TIFN41 and TIFN42A. The TIFN42A primer was designed as such that the original RBS plus the first two codons of the *spoVAA* gene were included in the amplified fragment. This was then cleaved with EcoRI and KpnI and introduced into the corresponding sites of pSG1151. The resulting pSGCVA vector was introduced into *B. cereus* ATCC 14579 and *B. cereus* ATCC 14579 Δ*spoVT* via electroporation and checked for single crossover integration on the original locus using PCR analysis and sequencing.

A strain bearing an in-frame deletion of the ORF encoded at locus BC1117 was created using a marker-less gene replacement method (Janes and Stibitz, 2006; Lindbäck et al., 2012). Essentially, a modified pMAD-derived vector containing ~500 bp of the respective up- and downstream regions of DNA flanking the BC1117 ORF was introduced by electroporation to *B. cereus* ATCC 14579 cells. Plasmid pBKJ233, which encodes I-SceI enzyme, was introduced subsequently to cells identified by blue-white (X-gal) screening and PCR as having inserted the pMAD-derived plasmid at locus BC1117. Clones that had undergone a second recombination event, deleting the BC1117 ORF and leaving behind only the start and stop codons, were identified by screening for erythromycin sensitivity. Finally, the ΔBC1117 strain was validated for the correct construction by PCR.

### Culture preparation and media

All *B. cereus* strains were cultured at 30°C with aeration at 220 rpm. Media was supplemented with chloramphenicol (4 μg/ml), erythromycin (2 μg/ml), or spectinomycin (300 μg/ml) when appropriate. For the preparation of cells for time-lapse microscopy, cells were prepared as previously described (Eijlander and Kuipers, 2013). For all other sporulation experiments and for the preparation of sporulating cells for RNA isolation, cells were cultured in maltose sporulation medium (MSM) (van der Voort et al., 2010). Spore crops were prepared on MSM agar plates for 7 days at 30°C. Spores were collected from the plates and washed (6000 rpm, 15 min at 4°C) twice a day for 14 days in 10 mM phosphate buffer (pH 6.2) with decreasing concentrations of Tween20 to prevent clumping of the spores (0.1, 0.075, 0.05, 0.04, 0.03, 0.02, and 0.01%). Phase contrast microscopy analysis of the spore crops showed >98% free-lying spores. Spores were stored at 4°C in 10 mM phosphate buffer with 0.01% added Tween20 and were washed twice a week to prevent spontaneous germination.

### Complementation of Δ*spoVT* with pNWVT (*spoVT*_BCE_) and pVTBsu2 (*spoVT*_BSU_)

The vectors pNWVT (containing *spoVT*_BCE_ expressed by its own promoter) and pVTBsu2 (containing *spoVT*_BSU_ expressed by the P_*spoVT*__BCE_ promoter) were introduced in the *B. cereus* ATCC 14579 Δ*spoVT* strain by electroporation as described above. Overnight cultures were diluted 1:100 in MSM medium and incubated at 30°C while shaking for 6 h. Exponentially growing cells were again diluted 1:100 in fresh MSM medium and allowed to grow in the same conditions for 65 h. Samples for phase contrast microscopy analysis were taken after 17, 22, 44, and 65 h. Efficiency in complementation of sporulation was determined using the Cell Counter plugin in ImageJ^1^.

### Germination assays

*B. cereus* spores were washed twice before the experiment and resuspended in ice-cold sterile demineralized water. Spores were heat-activated at 70°C for 15 min and immediately placed on ice for 2 min to cool down. The washing step was repeated after which spores were resuspended in ice-cold sterile germination buffer (10 mM Tris-HCl 7.4 + 10 mM NaCl) to a final OD_600_ of 10. Spores were diluted 10 times in germination buffer containing nutrients (10 mM alanine or 1 mM inosine) or BHI medium supplemented with chloramphenicol (5 μg/ml) to prevent outgrowth. Changes in optical density were monitored every 2 min for 4 h at 30°C in a TECAN plate reader and the obtained data plotted in Excel.

### Imaging, time-lapse microscopy, and image analysis

All microscopy imaging was performed using the IX71 Microscope (Olympus) with CoolSNAP HQ2 camera (Princeton Instruments) and DeltaVision softWoRx 3.6.0 (Applied Precision) software. For *B. subtilis* the 100x phase contrast objective was used, for *B. cereus* the 60x phase contrast objective. For the visualization of green fluorescence from GFP or FM46 dye the GFP filterset was used (Chroma, excitation at 470/40 nm, emission at 525/50 nm). Images were taken using 32% APLLC White LED light and 0.05 s exposure for bright field pictures and 10% Xenon light with 0.5 s exposure for fluorescence detection. Pictures were analyzed using ImageJ software.

To monitor gene expression in single cells during sporulation, the time-lapse microscopy technique was applied for the promoter-*gfp* fusion strains of *B. cereus* as previously described (Eijlander and Kuipers, 2013). For quantification of the GFP fluorescence signal distribution, the fluorescence intensity was measured for every cell in the microcolony for every time frame using the ImageJ ROI tool. Fluorescence intensities per cell per frame were exported to Excel and normalized by subtracting background fluorescence levels of the microscopy slide (agarose) and autofluorescence of the cells or spores (highest fluorescent value measured for wt strains). Mean fluorescence values were calculated from ten frames per cell and binned according to set fluorescence value categories. This data was represented in fluorescence distribution plots in Excel.

### Total RNA extraction from sporulating *B. cereus* cells

Cells were cultured in sporulation media to induce the sporulation event. Individual stages in sporulation were determined via fluorescence microscopy on agarose patches with added FM® 1–43 membrane stain (Invitrogen, Ex 479 nm, Em 598 nm) at an end concentration of 1 μg/μl.

Cell samples (5 ml, including biological and technical replicates) were taken every hour for 6 h from transition point onwards (T0, reached after 7 h of growth in MSM). Cells were collected by centrifugation in a pre-cooled centrifuge (4°C, 4000 rpm, 3 min) and immediately frozen in liquid nitrogen. For RNA extraction, cells were thawed on ice in 500 μl TRI-reagent (Life Technologies, Carlsbad, CA USA) and glass beads (<100 μm, Sigma-Aldrich), resuspended and immediately disrupted by 4 rounds of bead beating [45 s at maximal settings (3450 rpm)] in a Mini-Beadbeater-16 (BioSpec products, Bartlesville, OK USA). Direct-zol RNA MiniPrep (Zymo Research, Irvine, CA USA) was used according to manufacturer's instruction for on column RNA purification. Residual chromosomal DNA was removed using the Ambion DNA-*free*™ kit (Life Technologies®, Thermo Fisher Scientific, USA). The RNA concentration was measured on a NanoDrop ND-1000 spectrophotometer. RNA quality was determined using an Agilent BioAnalyzer RNA 6000 nanokit. The concentration of RNA isolated from wt sporulation cells was a little lower than for Δ*spoVT* sporulating cells (3000 ng/μl compared to 3800 ng/μl) but still comparable. Quality of all isolations was within the specified levels for all samples (an OD_260/280_ ratio between 1.8 and 2.0 and an OD_260/230_ ratio of >1.7).

### RNA sequencing

RNA samples (> 1 μg) were sent to PrimBio Research Institute (Exton, PA, USA) where rRNA depletion (using the Ambion MICROBExpress™ Kit) and a library prep (using the Ion Total RNA-Seq Kit v2) were performed. Samples were multiplexed in sets of nine and loaded on an Ion Proton™ chip. In total 80 M reads with an average length of 120 bases were derived per chip. The reads were mapped against the reference genomes for *B. cereus* ATCC 14579 using Bowtie 2 (Langmead and Salzberg, 2012) with optimized parameters setting for Ion Proton data: “-D 20 -R 3 -N 1 -L 20 -i S, 1, 0.50 –local”. Gene expression profiling was done using SAMtools (Li et al., 2009) and Cufflinks (Trapnell et al., 2012). Subsequent statistical analysis was performed using a modified EdgeR routine on the MolGen webserver^2^.

Spearman Rank correlation analysis was performed to show clustering of biological replicates within one strain and between strains. Correctly clustered data sets were further filtered based on intra-variation < inter-variation. Excel conditional formatting was applied to visualize the degree of intra- and inter-variation for each differentially expressed gene. The biological ratio in gene expression was calculated using log-transformed data in the following formula:

$$\begin{array}{l} Biological\;ratio=log2\left(wt1\;+\;wt2\right)-log2\left(dVT1\;+\;dVT2\right) \end{array}$$

where *wt*1 and *wt*2 are replicate rpkm values for the *wt* strain and *dVT*1 and *dVT*2 are replicate rpkm values for the Δ*spoVT* strain. The fold change in gene expression between the two strains was furthermore determined by raising the number 2 to the power of the absolute value for the ratio (2^ABS(ratio)^).

Orthologs of *B. subtilis* sporulation genes (SporeWeb^3^, Eijlander et al., 2014) in *B. cereus* ATCC 14579 were determined using OrthoMCL (Chen et al., 2006) and (predicted) gene function using the KEGG database^4^ or NCBI Blast^5^.

## Results

### SpoVT is essential for completion of sporulation in *Bacillus cereus*

**Figure 2:**
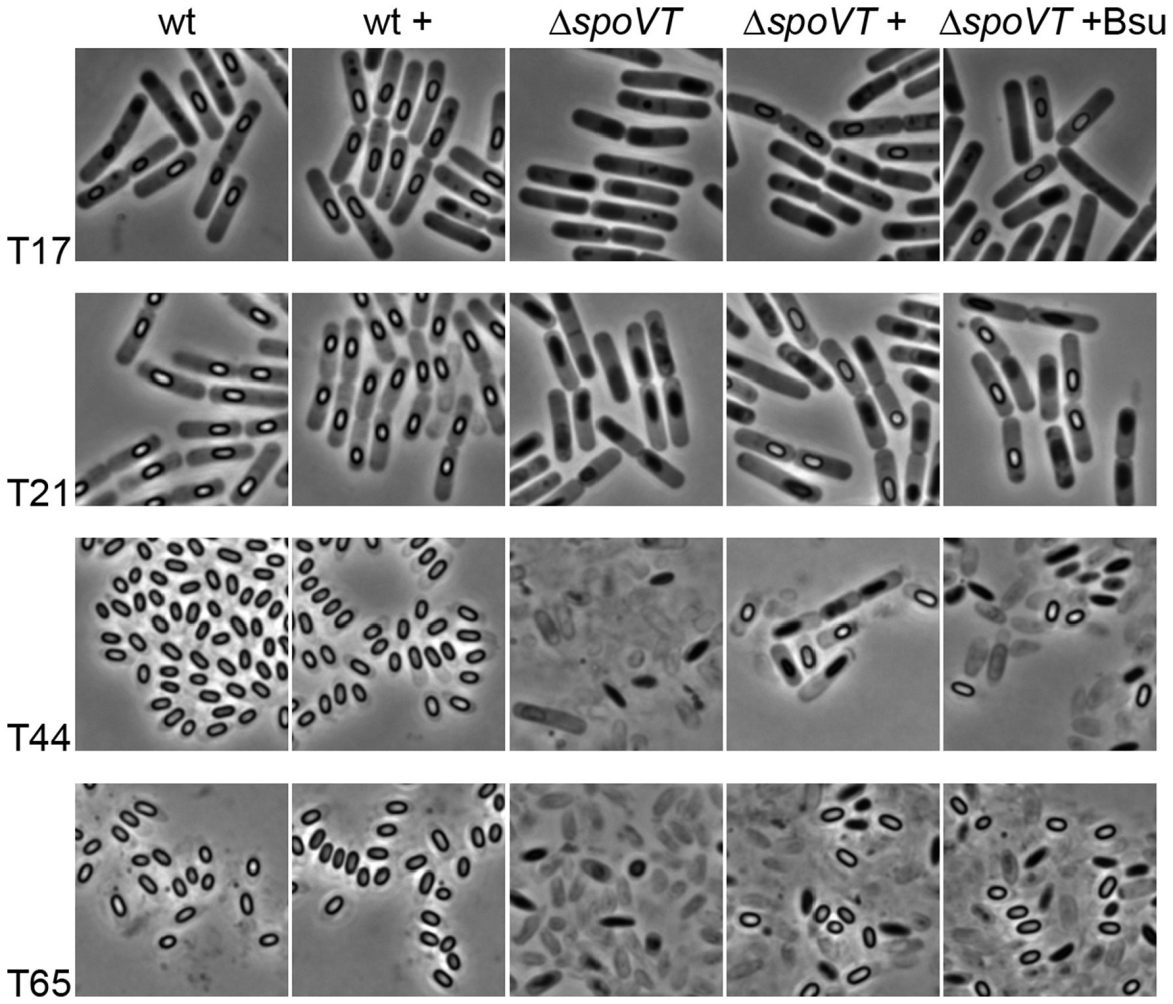
**Sporulation of ***B. cereus*** in the presence and absence of SpoVT**. Sporulation was induced by growth in MSM medium and the progress of sporulation was monitored using phase-contrast microscopy for the *B. cereus* ATCC 14579 wild type strain (wt) and *spoVT* deletion strain (Δ*spoVT*) with plasmid-borne *spoVT*_BCE_ (+) or *spoVT*_BSU_(+Bsu) (originating from the pNWVT or pVTBsu2 vector, respectively). The *B. cereus* wt strain with extra plasmid-borne *spoVT*_BCE_ (wt +) was added as a control to ensure no unexpected sporulation defects were occurring due to pNWVT-derived *spoVT* expression during sporulation. Samples were analyzed after 17, 21, 44, and 65 h of growth after initial dilution in fresh medium.

All present knowledge on the SpoVT regulator protein so far originates from studies in *B. subtilis*. Cells of *B. subtilis* that lack SpoVT are still able to produce spores, albeit with a defective spore coat structure, an increased initial germination rate in response to nutrients and a severe defect in further germination and outgrowth (Bagyan et al., 1996; Ramirez-Peralta et al., 2012). Despite a strong conservation of SpoVT in endospore-forming bacteria, the effect of removing SpoVT from *B. cereus* cells (SpoVT_BCE_) proves more severe. In this organism, a similar *spoVT* disruption mutant is unable to complete the sporulation process and produces very few, if any, free-lying spores with a severely defective morphology (Figure 2 Δ*spoVT*). Re-introduction of *spoVT*_BCE_ only partly restored this defect (14% of the total number of cells restored sporulation, Figure 2 Δ*spoVT*+). The partial complementation result could be due to differences in plasmid copy number between individual spores. Interestingly, similar complementation experiments with the *spoVT* wt allele of *B. subtilis* (*spoVT*_BSU_ driven by the P*spoVT*_BCE_ promoter) produced comparable results (complementation restored sporulation in 12% of the total number of cells, Figure 2, Δ*spoVT* +Bsu). This indicates that the working mechanism of SpoVT_BSU_ and SpoVT_BCE_ is comparable, which is expected given the high sequence conservation between both proteins. The observed difference in phenotypic effect after removal of SpoVT in both organisms must thus have a basis in the promoters of these regulatory proteins interact with to modulate gene expression.

### Sporulation of a *spoVT* deletion strain of *B. cereus* is stalled at a later stage leading to premature lysis

Time-lapse fluorescence microscopy is a powerful technique to visualize dynamics in gene expression in time in individual cells and was recently optimized for application with *B. cereus* cells (Eijlander and Kuipers, 2013). In this study, we applied time-lapse fluorescence microscopy to follow the events of forespore-specific gene expression in time in a *spoVT* deletion background of *B. cereus*. Unfortunately, multiple attempts to construct a variety of sporulation-specific promoter-*gfp* fusions (e.g., P_*gerI*_, P_*cwlJ*_, P_*gerR*_, P_*cotD*_, P_*sspA*_) stably integrated into the *B. cereus* genome failed, except for one (i.e., P_*spoVA*_-*gfp*). This limited our ability to determine true effects of SpoVT removal on the expression levels of late sporulation genes, but did enable us to provide insights in the sporulation line of events in the absence of SpoVT.

**Figure 3:**
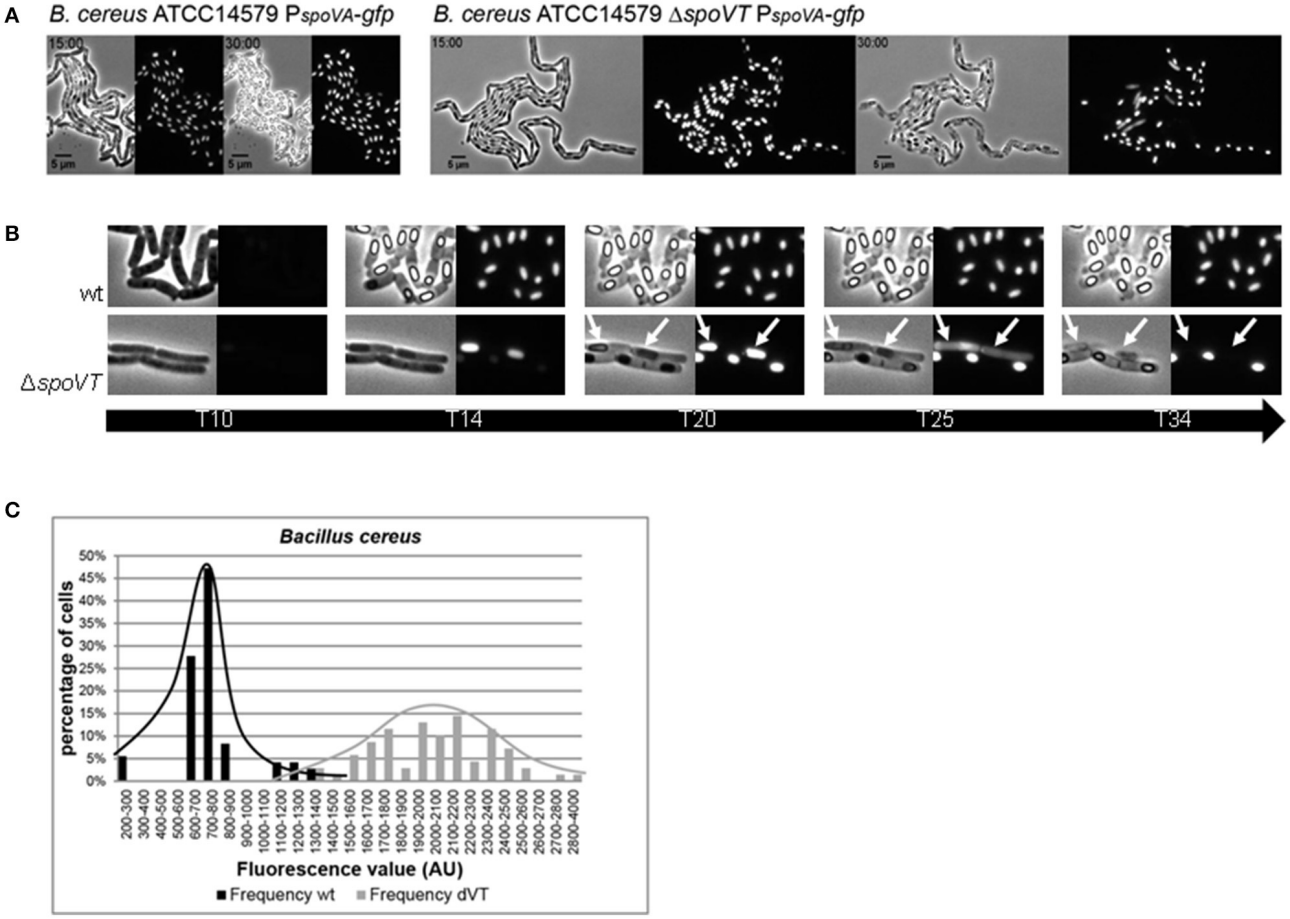
**P_***spoVA***_ promoter activity and signal distribution in a wt and ***spoVT*** deletion strain of ***B. cereus*****. P_*spoVA*_ promoter activity was measured in the wild type strain (*B. cereus* ATCC14579) and a Δ*spoVT* background using time-lapse microscopy. **(A)** Two time frames from movies S1 (wt) and S2 (Δ*spoVT*) show the expression of the P_*spoVA*_-*gfp* fusion during sporulation in individual cells. The time (in hours) is indicated in the top left corner. The scale (in μm) is indicated at the bottom left corner. **(B)** Cut-outs of individual cells during five different time points (in hours) from Movies S1, S2 are shown to visualize the line of events during sporulation in a wt and a Δ*spoVT* background. Lysing prespores and mothercells in Δ*spoVT* are indicated by white arrows. **(C)** The average fluorescence value distribution in arbitrary units in *B. cereus* wt (black bars) and *spoVT* cells (gray bars) calculated from Movies S1, S2.

Time-lapse image analysis of strain *B. cereus* Δ*spoVT* P_*spoVA*−__BCE_-*gfp* (Movie S2) clearly shows that during the earlier stages of sporulation prespores are formed like in the wild type background (Movie S1), but that progression of sporulation is stalled at a later stage. First, the formed pre-spores lyse, thereby spreading the produced GFP throughout the mother cell (Figure 3B and Movie S2). Finally, the mother cell also lyses (Figure 3A).

A quantitative analysis of the obtained images was performed to substantiate the difference in *spoVA* gene expression levels in a *spoVT* background. Before pre-spore lysis, the intensity of fluorescence originating from the P_*spoVA*_-*gfp* fusion was significantly higher in the absence of SpoVT_BCE_ than in wild type cells (Figure 3C and Movies S1, S2). In addition, the distribution of the signal strength was more wide-spread amongst individual cells. It must be noted, however, that it is possible that this observed effect is not the result of increased (heterogeneity in) promoter activity in the absence of SpoVT, but rather an artifact in the detection of the GFP signal—the *spoVT* spores are severely weakened shortly after the completion of engulfment and, furthermore, it is known that SpoVT enhances rather than represses *spoVA* expression in *B. subtilis* (Wang et al., 2006; Ramirez-Peralta et al., 2012).

### Transcriptomic analysis of *B. cereus* Δ*spoVT* during sporulation

To further investigate the role of SpoVT in gene expression during sporulation of *B. cereus*, we isolated RNA from sporulating cells at three sequential time points (3, 4, and 5 h after the transition point to stationary growth was reached; Figure S1). Differentially expressed genes in the *spoVT* deletion strain were compared to the wild type situation using a transcriptomics approach. Details on RNA isolation, preparation, sequencing, downstream normalization, and statistical analysis of the resulting data are described in the Materials and Methods Section. Resulting normalized expression values [reads per kilobase per million (rpkm)] are provided in Table S2.

Sporulation is a very heterogeneous process, which complicates the synchronization of gene expression in individual cells. This generates a large variety in the absolute gene expression values generated by RNA sequencing. Spearman Rank correlation of the available data showed that two biological replicates of the same strain (wt or Δ*spoVT*) clustered together only for the samples taken at T5. Therefore, further data analysis was performed for that time point only.

The log2-transformed data was used to calculate the fold change in gene expression in the *spoVT* mutant strain compared to the wt strain. A total list of significantly up- and down-regulated genes is provided in Table S3.

Of the differentially expressed genes (with a cut-off of ≥2-fold difference), a total number of 52 genes were down-regulated and 87 genes were up-regulated (Table 2). Both groups contain genes involved in sporulation, germination, regulation, metabolism, and transport and both groups contain a large number of genes encoding hypothetical or unknown proteins. What stands out, is that genes involved in protein synthesis (ribosomal protein subunits) were specifically upregulated in a *spoVT* mutant. In addition, a large number of genes involved in metabolism was also upregulated.

**Table 2:** **Number of genes per functional category up-or down-regulated in a ***B. cereus spoVT*** deletion background**.

**Functional category**	**Number of genes upregulated**	**Number of genes downregulated**
Sporulation	15	9
Germination	4	1
Metabolism and transport	25	10
Protein synthesis	13	0
Regulation	6	4
Hypothetical and unknown	16	20
Other	8	8

Concerning sporulation and/or germination-specific genes, almost no *B. cereus* orthologs of known SpoVT-regulated genes (as determined by studies in *B. subtilis*; Bagyan et al., 1996; Wang et al., 2006) are present in this list. Only *spoIIIG* (BC3903) and *tepA* (BC3795) were, similarly to their counterparts in *B. subtilis*, repressed by SpoVT in *B. cereus* (Table S3). Amongst significantly down-regulated sporulation genes (>2-fold difference in gene expression) were some genes encoding spore coat proteins, one germination receptor subunit (GerIA) and a few sporulation proteins of unknown function. Upregulated sporulation genes, on the other hand, were those encoding CwlJ1, SpoIIQ, SpmA, CotD, YaaH, SpoIVA, SpoIIIAH, CdaS, SpoIIIAC, genes encoding sporulation-specific regulators RsfA, SigK, GerE, SigG, and AbrB and a few sporulation proteins of unknown function.

Interestingly, quite a few hypothetical or genes of unknown function were extremely, or at least considerably, downregulated in the *spoVT* deletion strain (Table 3). Although no putative function could be deducted from homology searches using their protein sequences, most of them seem to be specific for *B. cereus* group organisms. One such example is the gene encoded at locus BC1117, expression of which was down-regulated approximately 3000-fold in the *spoVT* mutant strain. BC1117 is predicted to encode a small (50 residue, ~6 KDa) lysine-rich polypeptide. A preliminary examination of the role of BC1117 in *B. cereus* ATCC 14579 was conducted by constructing a strain with a marker-less deletion in this locus. However, in contrast to the Δ*spoVT* strain, the ΔBC1117 strain was observed to grow and sporulate normally, releasing mature spores after approximately 24 h of culture (Figure S2).

**Table 3:** **Significantly downregulated hypothetical genes in a ***B. cereus spoVT*** deletion mutant**.

**Gene locus tag**	**Fold-change (down-regulated)**	**Protein homology**
BC1117	2984	Hypothetical protein, homology to *B. cereus* group-specific hypothetical protein
BC0987	37	Hypothetical protein, homology to hypothetical protein
BC1457	20	Hypothetical protein, homology to hypothetical protein
BC2492	18	Hypothetical protein, homology to *B. cereus* group-specific hypothetical protein
BC2270	15	Hypothetical protein, homology to *B. cereus* group-specific hypothetical protein
BC3002	10	Hypothetical protein, homology to *B. cereus* group-specific hypothetical protein
BC0973	9	Hypothetical protein, *B. cereus* group membrane protein
BC2426	8	Hypothetical protein, homology to *B. cereus* group-specific hypothetical protein

## Discussion

SpoVT is a strongly conserved regulatory protein specifically active during the later sporulation stages in the forespore compartment of cells and is known to play an important role in fine-tuning the regulation of sporulation-specific gene expression (Ramirez-Peralta et al., 2012). The lack of a consensus SpoVT binding sequence complicates the prediction of alternating SpoVT level effects on spore properties and spore germination efficiency. The strongly conserved N-terminal DNA-binding domain of SpoVT suggests a similar conservation in DNA targets for this regulatory protein. However, in this work we show that removing SpoVT from sporulating cells has a dramatic effect on completion of sporulation in *B. cereus*—an effect that is not observed in *B. subtilis*. Unfortunately, we were unable to study single copy integrated SpoVT complementation due to the low efficiency of DNA recombination in *B. cereus* (Arnaud et al., 2004). By making use of a vector-based complementation, restoration of the sporulation phenotype was only partly accomplished. This could be due to differences in plasmid copy number between individual spores (Turgeon et al., 2008), which would suggest that the tolerated levels of SpoVT_BCE_ protein in sporulating cells fit inside a tight window.

Furthermore, similar complementation results with heterologous SpoVT_BSU_ indicate that the observed difference in the impact of SpoVT removal from sporulating cells of the two species is not due to a difference in the working mechanism, but rather to a difference in regulated target genes. This is supported by transcriptomic analysis of differential gene expression during late-stage sporulation, in which a significant number of *B. cereus*-group specific genes of unknown function were severely down-regulated in the *spoVT* mutant strain. The potential role of one of these genes (BC1117) in sporulation was further investigated using a clean BC1117 deletion mutant strain. This strain sporulated normally, indicating that the sporulation phenotype observed in the Δ*spoVT* strain cannot be solely attributed to the extreme down-regulation of the BC1117 gene. The function of BC1117 and whether it plays a direct or indirect role in sporulation remains to be elucidated. Work is continuing to further characterize the BC1117 polypeptide in terms of its location and role in *B. cereus* spores.

Unfortunately, the synchronization of sporulation for RNA isolation proved challenging, limiting the usability and reliability of the obtained RNA sequencing data sets to time point T5 only. This could be the reason why only a limited number of known SpoVT regulon members were represented in our data set, which does not allow us to define the SpoVT regulon in *B. cereus*. Potentially, the optimization of sporulation protocols for *B. cereus* cells can help overcome this issue.

What can be derived from the transcriptomic data, however, is that the observed sporulation defect caused by lysis of formed prespores in *B. cereus spoVT* might be the result of premature germination of the prespores. Genes involved in both metabolism and protein synthesis were upregulated especially in the *spoVT* deletion strain, which is known to occur during the early stages of germination (Horsburgh et al., 2001). In addition, the spore cortex lytic enzyme (SCLE) CwlJ1 and the cortical fragment lytic enzyme (CFLE) SleL were upregulated over 5-fold and almost 4-fold, respectively, in a *spoVT* mutant of *B. cereus*. CwlJ is responsible for the degradation of the cortex layer upon germination and responds specifically to exogenous Ca^2+^-chelated dipicolinic acid (Ca-DPA) (Moir, 2006), whereas SleL cooperates with CwlJ by hydrolysing fragmented peptidoglycan substrates (Lambert et al., 2012), which supports the theory of premature germination in *B. cereus* Δ*spoVT*. Both genes encoding these proteins are known to be expressed in the mother cell under the control of σ^E^ and SpoIIID during early stages of sporulation. This suggests that the observed upregulation in a *spoVT* deletion mutant is more than likely an indirect effect. Such indirect effects may be more abundant, as several other regulatory proteins were also upregulated in the *spoVT* deletion strain (RsfA, SigK, GerE, and SigG). Together with the lack of a consensus SpoVT binding sequence, this complicates the determination of the SpoVT regulon in *B. cereus*.

The heterogeneous character of SpoVT in sporulating bacteria is intriguing. Through this study we demonstrate an essential role of the SpoVT protein in sporulation of *B. cereus*, whereas in *B. subtilis* SpoVT is not essential, but nevertheless very important for proper formation and maturation of a spore. Further investigation on the essential and/or fine-tuning role of SpoVT in the regulation of sporulation-specific gene expression requires the quantification of active SpoVT levels, preferably in individual cells. This is currently complicated by the gaps in our knowledge on possible SpoVT activity requirements, *e.g*., the binding of a (yet unidentified) substrate. Obtaining such knowledge will also provide us with more insights on the role of SpoVT in sporulation and germination heterogeneity. Such phenotypic heterogeneity is an outcome of bifurcation in gene regulatory circuits, in which key regulatory proteins are often subjected to multimerization and require additional modification for activation before instigating feed forward loops in gene expression. In *B. subtilis* developmental processes, Spo0A and ComK are well-studied examples of such regulatory proteins (Grossman, 1995; Lopez et al., 2009). Given the requirement of tetramerization and possible substrate binding prior to activation, it is very plausible that SpoVT is equally involved in such complex regulatory circuits and is thus one of the major players responsible for germination heterogeneity. Levels of such a substrate may differ per individual cell, especially if the regulation of its production is part of a signal transduction system. Unfortunately, numerous attempts to identify such a substrate have thus far failed (Dong et al., 2004; Asen et al., 2009). New leads can be defined based on novel findings on the production of signaling molecules during sporulation, such as the recent demonstration of spore-specific production of cyclic di-AMP (c-di-AMP) *via* the CdaS cyclase during late-stage sporulation (Mehne et al., 2013, 2014). Since the absence of CdaS in sporulating *B. subtilis* cells results in an affected germination profile of resulting spores (Mehne et al., 2014) and since *cdaS* deletion has a negative (direct or indirect) effect on sporulation gene expression levels (qPCR data not shown), it is tempting to assume that CdaS-derived c-di-AMP functions as a trigger molecule for SpoVT activity. Unfortunately, we have so far been unsuccessful in demonstrating an *in vitro* interaction between c-di-AMP and purified SpoVT_BSU_, whereas a clear interaction of c-di-AMP with the positive control DarA was observed (Jörg Stülke, personal communication, data not shown). Possibly, forespore-specific interactions between molecules are extra challenging to simulate *in vitro* (Asen et al., 2009), or c-di-AMP plays an important role during spore germination and/or outgrowth not related to SpoVT activity.

Differences in regulons of conserved regulatory proteins are not uncommon. In *B. thuringiensis* for instance, an organism closely related to *B. cereus*, it was found that the SinR regulatory protein was not involved in the regulation of an *eps* operon like in *B. subtilis*, but rather in the biosynthesis of the lipopeptide kurstakin and the Hbl enterotoxin gene that is not part of the *B. subtilis* genome (Fagerlund et al., 2014). In addition, for clostridial species it is well known that the regulatory programs orchestrating sporulation differ significantly from those in *B. subtilis* despite strong regulator protein conservation (Al-Hinai et al., 2015).

In summary, this report further demonstrates that despite conservation in sporulation regulatory proteins amongst Bacilli (de Hoon et al., 2010), future studies on their function and impact should take into account evolutionary differences in regulon members between species underlying important variances in downstream developmental processes.

## Author contributions

RE designed the experimental plan, executed the majority of the experiments, analyzed the results, and wrote the manuscript. SH supported in the technical part and read the final manuscript. AD analyzed the transcriptomic results, contributed in writing, and read the final manuscript. AG executed the experiments with the BC1117 mutant, contributed in writing, and read the final manuscript. GC supported in the analysis of the BC1117 mutant results, contributed in writing, and read the final manuscript. OK participated in the experimental plan and read the final manuscript.

## Funding

This research is funded by TI Food and Nutrition, a public-private partnership on pre-competitive research in food and nutrition. The funders had no role in study design, data collection and analysis, decision to publish, or preparation of the manuscript.

### Conflict of interest statement

The authors declare that the research was conducted in the absence of any commercial or financial relationships that could be construed as a potential conflict of interest.

## Footnotes

^1^http://imagej.nih.gov/ij/.

^2^http://genome2d.molgenrug.nl, T-REx DOI: 10.1186/s12864-015-1834-4.

^3^http://sporeweb.molgenrug.nl.

^4^www.genome.jp/kegg/.

^5^http://blast.ncbi.nlm.nih.gov/.

## Supplementary material

Figure S1 **Fluorescence microscopy analysis of cell sampling for transcriptomic analysis**. The sequential stages in sporulation were monitored in time using a fluorescing membrane dye. Time points (in hours) after initiation of sporulation are indicated.

Figure S2 **Light microscopy analysis of ***B. cereus*** ATCC 14579 cultures approximately 24 h after entry to sporulation**. Mature spores are evident in the **(A)** wild type and **(C)** ΔBC1117 cultures, whereas the ΔSpoVT cells **(B)** have arrested during sporulation. Scale bar indicates 5 μM.

Table S1 **Primers used in this study**.

Table S2 **Gene expression during sporulation in ***B. cereus*** and ***B cereus*** Δ***spoVT*****. Duplicate gene expression values during three time points in sporulation (T3, T4 and T5) for the wild type (WT) and the spoVT deletion strain (delta-VT) are indicated as reads per kilobase per million (rpkm).

Table S3 **Significant up-and downregulated genes in a ***spoVT*** deletion background of ***B. cereus*** during T5**. Down-regulation in gene expression in the *spoVT*-mutant strain is visualized in red, whereas up-regulation is visualized in green. The data for two biological replicates of the same strain are shown separately. The ratio in gene expression was calculated by taking the difference of the 2log-transformed values at T5 between the wt and the spoVT mutant strain. The fold change in gene expression was calculated by raising the number 2 to the power of the absolute ratio value. Orthologs of known sporulation genes in *B. subtilis* are indicated in bold face. Regulation during sporulation in *B. subtilis* was derived from information on SporeWeb http://sporeweb.molgenrug.nl).

Movie S1 **Time-lapse microscopy movie for sporulating wild type ***B. cereus*** cells**. The fluorescence shown in the right panel is derived from GFP expression driven by the P_*spoVA*_ promoter.

Movie S2 **Time-lapse microscopy movie for sporulating cells of *B. cereus* delta-*spoVT***. The fluorescence shown in the right panel is derived from GFP expression driven by the P_*spoVA*_ promoter.
