# Supplementary figures and images for: SpoVT: From Fine-Tuning Regulator in Bacillus subtilis to Essential Sporulation Protein in Bacillus cereus
Source: Front Microbiol. 2016 Oct 13;7:1607. doi: 10.3389/fmicb.2016.01607 (PMC5061766; doi:10.3389/fmicb.2016.01607)

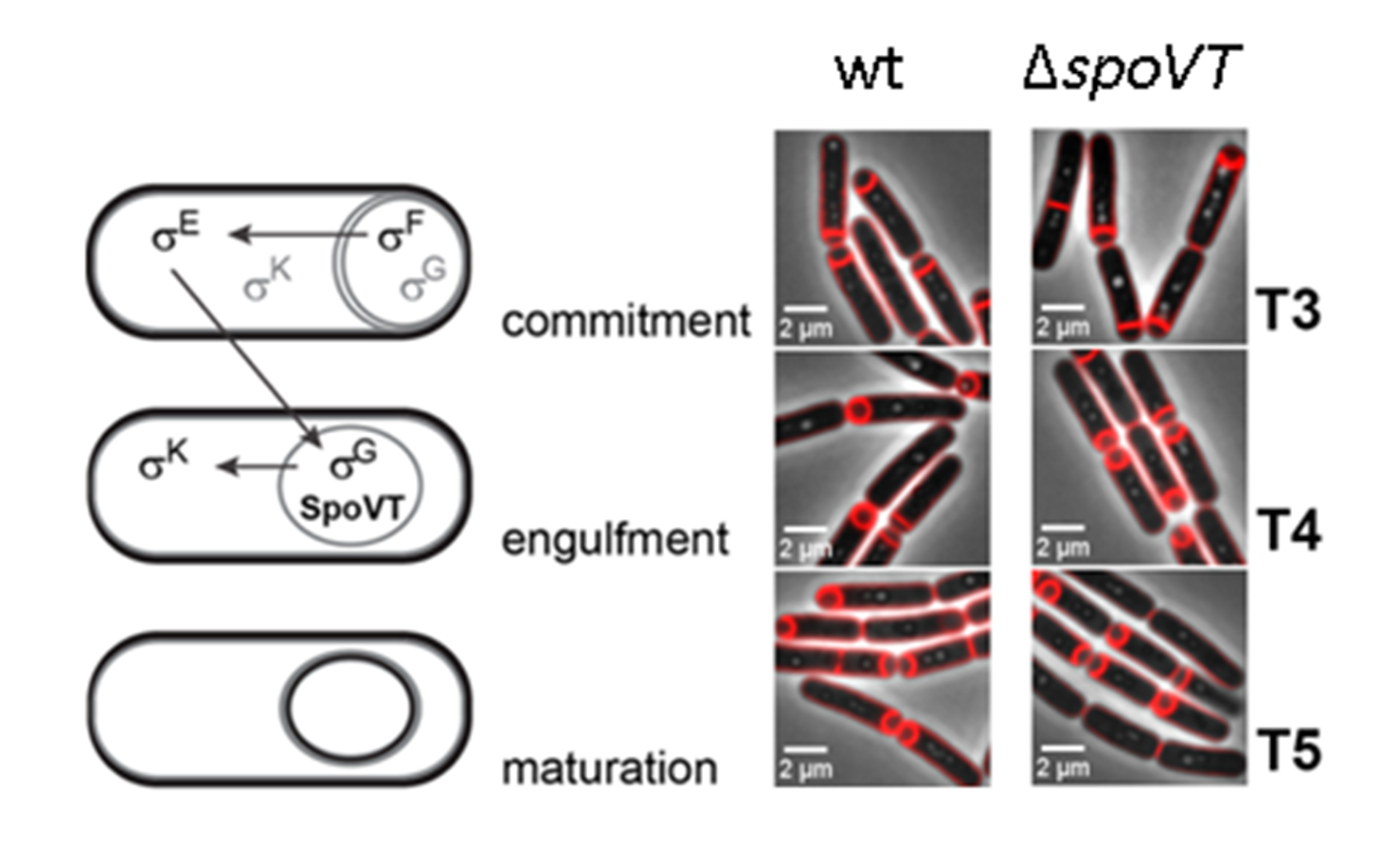

Supplement: Figure S1 — Fluorescence microscopy analysis of cell sampling for transcriptomic analysis. The sequential stages in sporulation were monitored in time using a fluorescing membrane dye. Time points (in hours) after initiation of sporulation are indicated. [file Image1.TIF]

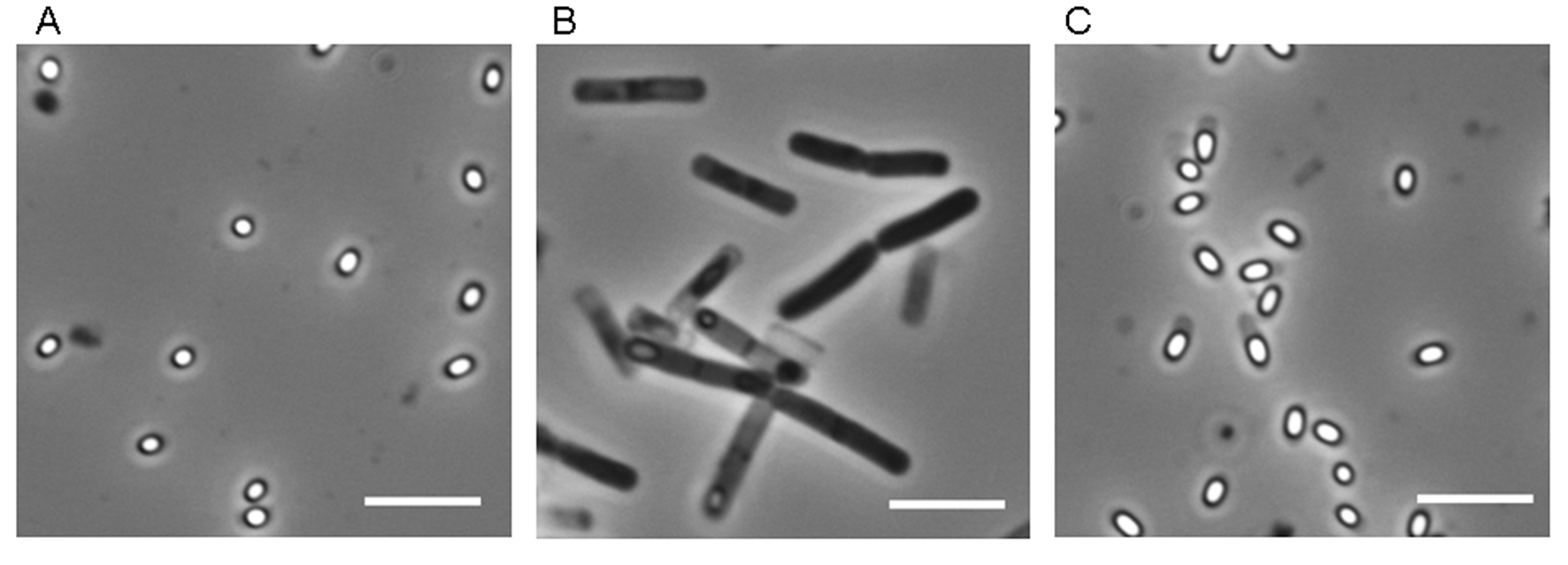

Supplement: Figure S2 — Light microscopy analysis of B. cereus ATCC 14579 cultures approximately 24 h after entry to sporulation. Mature spores are evident in the (A) wild type and (C) ΔBC1117 cultures, whereas the ΔSpoVT cells (B) have arrested during sporulation. Scale bar indicates 5 μM. [file Image2.TIF]
